# Attitudes, beliefs, and knowledge regarding medical cannabis among healthcare students in the Republic of Cyprus: a cross-sectional descriptive correlational study

**DOI:** 10.3389/fpsyt.2023.1196915

**Published:** 2023-07-14

**Authors:** Sokratis Sokratous, Meropi Mpouzika, Katerina Kaikoushi, George Alexandrou, Maria Karanikola

**Affiliations:** ^1^Department of Nursing, School of Health Sciences, Cyprus University of Technology, Limassol, Cyprus; ^2^Cyprus Mental Health Services, Larnaca, Cyprus

**Keywords:** Cyprus, healthcare students, attitudes, beliefs, knowledge, medical cannabis

## Abstract

**Background:**

Although international research-based literature from the last 2 decades seems to favor the use of medical cannabis (MC), there is a lack of evidence concerning healthcare students’ education on MC in the Republic of Cyprus and across the world. Therefore, this study explores healthcare students’ attitudes, beliefs, and knowledge regarding the use of MC. We paid special attention to differences across specific sociodemographic (gender, age, and religion status) and educational (level of study and study field) characteristics.

**Methods:**

A descriptive cross-sectional study was conducted between November 2019 and March 2020. All active undergraduate and postgraduate healthcare students (nurses, physiotherapists, speech therapists, pharmacists, and occupational therapists; *N* = 900) studying in public and private universities in the Republic of Cyprus were eligible to participate (final sample: *N* = 819, response rate = 91%). To collect data on the attitudes, beliefs and knowledge of the participants, we used the Medical Cannabis Questionnaire (MCQ). To analyze the data, we employed the Pearson’s chi-square test for group differences, in addition to assessing the descriptive and inferential statistics.

**Results:**

Approximately 82.2% believed that MC education should be integrated into the clinical practice requirements. Statistically significant differences were observed between genders in terms of beliefs/risk associated with the use of MC, with males being more likely to believe that there are significant mental-health benefits associated with using ΜC compared to females (84.9% vs. 76.2%, *p*<0.05). Females were more likely than males to believe that using MC poses serious physical (76.8% vs. 60.6%, *p*<0.001) and mental-health (77.9% vs. 66%, *p*<0.001) risks. Moreover, participants who received formal education about MC during their study/training were more prepared to answer patient/client questions about ΜC (*p* < 0.001). In addition, participants who received formal education had more frequently friends (*p* < 0.001) or family members who used MC (*p* < 0.005).

**Conclusion:**

This study provides useful information for curriculum development, educational changes, and policy decisions related to cannabis use for medical purposes in the Republic of Cyprus. The results showed that the majority of the healthcare students who participated in the study favored MC use. However, the participants reported a lack of knowledge and recommended additional evidence-based research and education to enhance their knowledge about MC use. Therefore, we recommend the implementation of formal education on MC among healthcare students in the Republic of Cyprus during their study and clinical training. Furthermore, it is important to include MC-related theoretical and clinical/laboratory courses during studies and clinical practice.

## 1. Introduction

The term cannabis refers to pharmacological agents derived from plants belonging to the genus Cannabis (1). Cannabidiol (CBD), a cannabis compound, is associated with multiple therapeutic benefits (2). According to international studies conducted over the last 2 decades, medical cannabis (MC) can be used in an effective medical treatment to manage the symptoms of chronic pain, anxiety, and severe and terminal illnesses (3), and can also be used as an alternative treatment for patients who do not respond to conventional medical interventions (2, 4, 5).

In 2019, legalized MC was used in the Republic of Cyprus (RC) after free prescription to eligible patients. Specifically, the parliament of the RC voted on a law that allowed the use of pharmaceutical cannabis in patients with chronic pain associated with cancer, neuropathy, rheumatism, HIV, and many other medical disorders (6). Thus, in the RC as in many other countries, physicians/healthcare professionals have the authority and responsibility to prescribe, recommend, and support non-pharmaceutical CBD products for therapeutic purposes in compliance with state laws (7). Additionally, healthcare providers (physicians, nurses, physiotherapists, etc.) play an important role in healthcare service users’ decision-making concerning MC for therapeutic use. Subsequently, healthcare professionals as educators and advocates, should have the skills and competencies to empower, inform and educate, and support the individuals they care for regarding MC related issues (8, 9). Nevertheless, healthcare providers should provide evidence-based information that has to tailored to the personalized needs of individuals, taking into account their beliefs, life-style and goals. Based on the above, healthcare students, as the future healthcare professionals, need to be comprehensively educated to be able to provide optimal care and treatment, in terms of decision-making and needs management, to those who will be legally allowed to use MC for therapeutic purposes. Yet, during the past few years, scholars have observed gaps in healthcare students’ education on MC (10–14).

At the same time, there is evidence that every day clinical practice is associated with the stereotypes, knowledge and personal beliefs that healthcare providers hold (15). Moreover, students’ personal beliefs and assumptions seem to influence their attitudes towards care when they became registered clinicians (16, 17). However, only few studies have been published on healthcare students’ knowledge, attitudes, and beliefs regarding MC. Additionally, the vast majority of such studies have been performed in countries where MC has been legalized; thus, there is a lack of information on the use of MC in countries where it is not authorized (18) or where it’s use is in the embryonic stage (e.g., RC).

### 1.1. Aim

This study explored the attitudes, beliefs, and knowledge regarding MC use among healthcare students (nurses, physiotherapists, speech therapists, pharmacists, and occupational therapists) in the RC. We paid special attention to differences across specific sociodemographic (gender, age, and religious status) and educational (level of study and study field) characteristics.

## 2. Methods

### 2.1. Design, setting, and participants

A descriptive cross-sectional study was conducted between November 2019 and March 2020. All active undergraduate and postgraduate nursing, physiotherapy, speech therapy, pharmacist, and occupational therapist students in the public and private universities, which comprise heath-allied study programs, were eligible to participate. There were no exclusion criteria in terms of age, gender, and nationality, as long as the participants to be were able to communicate in the Greek language, in which the questionnaire was developed. A total of 900 students were approached. Specifically, one out of three public universities (*n* = 350 students) and two out of four private universities (*n* = 550 students) agreed to take part to the study. Medical and psychology students were not included in the present sample, since relevant faculties did not respond to the call for participation in the study.

### 2.2. Data collection

Data collection took place in the students’ classrooms. Participation in the study was voluntary and anonymous to ensure confidentiality. Questionnaires and consent forms were distributed among the healthcare students at the beginning of the lecture. After a short briefing on the aims and procedures of the study, the healthcare students who wished to participate were asked to place their filled-in questionnaires in sealed envelopes in a collection box located outside the lecture room.

The study was approved by the National Bioethics Committee (Ref. No 2019.01.155) and the research committee of the participating universities.

### 2.3. Instruments

The Medical Cannabis Questionnaire (MCQ) was used for data collection on students’ attitudes, beliefs, and knowledge about MC (19, 20). The questionnaire was developed for cross-national studies on MC education among healthcare professionals and students (20–26). Thirteen items of the MCQ assessed attitudes and beliefs toward MC/cannabis (e.g., benefits, risks, and effectiveness). Eighteen items assessed beliefs and knowledge about the effectiveness of MC on medical conditions, whereas two items assessed beliefs and attitudes regarding MC education. Educational training-related attitudes toward MC were assessed using two items with predefined answers. One item assessed the participants’ attitudes towards formal and informal sources of information on MC. The MCQ has a high level of internal consistency reliability (Cronbach’s alpha values ranging from 0.767 to 0.831) (20, 24).

The MCQ was translated, back translated and validated in a previous study on the attitudes, beliefs, and knowledge regarding MC among Greek-Cypriot nursing students, and exhibited a high level of internal consistency reliability (Cronbach’s alpha values ranging from 0.75 to 0.85) (21).

The data collection instrument also included a section with variables on demographic characteristics (age, gender, religion, origin, family status, and employment status), educational level (years of study, highest degree completed, field of expertise, and years of work experience), and MC-related behaviors.

### 2.4. Statistical analysis

Descriptive statistics were calculated for the socio-demographic characteristics and the MCQ items, and were expressed as frequencies, mean values, and standard deviations. Responses to the ordinal MCQ variables were grouped into the following three categories: (a) agree/effective, (b) disagree/ineffective, and (c) do not know. Differences between the groups were assessed according to gender, age, religion, field, and level of study using the Pearson’s chi-square test. Multivariable backward stepwise logistic regression analysis, after adjusting for sociodemographic characteristics, was performed to confirm our results. SPSS (version 25.0) statistical software was used for data analysis. The significance level was set at α = 0.05.

## 3. Results

### 3.1. Socio-demographic characteristics

The sample (*n* = 819, response rate: 91%) consisted of nursing (*n* = 253), physiotherapy (*n* = 275), speech therapy (*n* = 112), and a total of 179 pharmacists and occupational therapy students. Among the non-participants (*n* = 81), 64 were students who were absent on the day of data collection, 13 students were present but refused to participate, and four students were excluded from the analysis due to missing or incomplete data. In total, 560 (68.4%) participants were male and 259 (31.6%) were female. The mean age of the participants was 21.48 years (SD: 4.07; range: 17–50 years). The vast majority of them were of Cypriot origin (*n* = 564, 68.9%), 168 (20.6%) were Greek, and 87 (10.5%) were foreigners. Most participants were Christian Orthodox (*n* = 744, 90.8%), while the rest reported other religions (*n* = 75, 9.2%). Concerning employment status, the majority of the sample were unemployed (*n* = 545, 66.5%); most participants were third-year (*n* = 236, 28.7%) and fourth-year (*n* = 212, 25.9%) students; while 767 (93.6%) were undergraduate students, 27 (3.3%) were master’s students, and eight were PhD students. Most participants were physiotherapy (*n* = 275, 33.7%) and nursing (*n* = 253, 30.8%) students (Table 1).

**Table 1:** Socio-demographic characteristics of the participants (*n* = 819).

Variables	*n* (%)
Mean age = 21.48 (Range 17–50 years/SD = 4.07)	
Gender	
Female	560 (68.4)
Male	259 (31.6)
Religious	
Christian orthodox	744 (90.8)
Other	75 (9.2)
Degree of loyalty	
Not religious	102 (12.5)
Somewhat religious	327 (39.9)
Religious/Very religious	390 (47.6)
Mother born	
Cyprus	564 (68.9)
Greece	168 (20.6)
Other	87 (10.5)
Family status	
Single	369 (44.9)
Other	450 (54.8)
Current employment status	
Full-time/Part-time employed	274 (33.5)
Unemployed	545 (66.5)
Academic status	
Undergraduate degree	767 (93.6)
Master degree	27 (3.3)
Doctorate of philosophy	8 (1.0)
Other post graduate level degree	17 (2.1)
Year of study	
First	173 (21.1)
Second	161 (19.7)
Third	236 (28.7)
Fourth	212 (25.9)
More	37 (4.6)
Field of study	
Nursing	253 (30.8)
Physiotherapist	275 (33.7)
Speech therapy	112 (13.6)
Other (Occupational therapist and pharmacist)	179 (21.9)

### 3.2. Participants’ attitudes and beliefs regarding MC

A vast majority of participants believed that healthcare professionals should have formal training related to MC before recommending it to patients (*n* = 766, 93.5%). Moreover, many participants (*n* = 673, 82.2%) believed that educational training in MC should be integrated into the practice and clinical practice requirements. At the same time, most participants believed that educational training in MC must be integrated into academic programs for all health-allied professionals (*n* = 772, 90.6%). Furthermore, the participants stated that physicians who prescribe MC should be in a continuous contact with the healthcare service users they care for (*n* = 794, 96.9%). Moreover, 84.9% (*n* = 696) of the participants noted that they would recommend MC to their patients, and eight out of 10 participants supported the idea that physicians should recommend cannabis for medical therapy (*n* = 675, 82.4%).

### 3.3. Participants’ attitudes, beliefs, and knowledge about MC regarding the treatment of specific medical conditions

Male compared to female participants believed more strongly that MC was effective for the treatment of a number of conditions, such as persistent muscle spasm (92.6 vs. 80%), terminal illness (90 vs. 81.9%), or mental health disorders (87.4 vs. 77.5%; *p* < 0.05; see Table 2 for more conditions). Additionally, the participants who were younger than 23 years compared with the participants who were older than 23 years believed more strongly that MC was effective in a number of conditions, such as AIDS/HIV (52.1 vs. 42.4%), while, the group of those aged older than 23 years believed more strongly that MC was effective for Parkinson’s disease than the other age groups (≥23 years old: 84.7% vs. 21–22 years old: 81% and 18–22 years old: 72.1%), (*p* < 0.05; see Table 2 for more information). Additionally, non-Cristian Orthodox group compared with the Christian Orthodox participants considered more frequently that MC was effective for a number of medical conditions (e.g., fibromyalgia/insomnia/sleeping disorders, Parkinson’s disease, etc.; *p* < 0.05); in terms of academic topic, nursing and physiotherapy participants expressed more frequently the belief that the use of MC is effective for specific medical conditions (e.g., persistent muscle spasm and insomnia/sleeping disorders) compared with other participants (e.g., speech therapy). Moreover, the vast majority of postgraduate participants considered MC to be effective in specific diseases compared to undergraduate participants (*p* < 0.05; Table 2).

**Table 2:** Association between socio-demographic characteristics and participants’ agreement regarding the effectiveness of MC on medical conditions.

	Gender				*p* value
Participants’ agreement on the effectiveness of MC on medical conditions	Female	Male			
	% (*n*)	% (*n*)			
Eating disorders	46.0 (186)	68.0 (115)			<0.001
Insomnia/Sleeping disorders	77.2 (355)	86.9 (186)			0.002
Mental health disorders	77.5 (366)	87.4 (194)			0.001
Nausea	44.5 (171)	56.3 (98)			0.006
Parkinson’s disease	73.5 (300)	87.0 (161)			<0.001
Persistent muscle spasm	80.0 (340)	92.6 (189)			<0.001
Epilepsy	70.7 (285)	81.9 (149)			0.003
Terminal illness	81.9 (348)	90.0 (162)			0.007
Alzheimer’s disease	67.8 (274)	76.8 (139)			0.017
	**Age**				***p* value**
**Participants’ agreement on the effectiveness of MC on medical conditions**	**18–20 years old**	**21–22 years old**	**>23 years old**		
	**% (*n*)**	**% (*n*)**	**% (*n*)**		
Nausea and/or vomiting due to cancer	65.9 (195)	71.3 (144)	78.7 (100)		0.027
AIDS/HIV	39.2 (96)	52.1 (86)	42.4 (39)		0.033
Insomnia/Sleeping disorders	73.8 (234)	84.2 (187)	88.9 (120)		<0.001
Mental health disorders	76.2 (253)	84.6 (187)	85.2 (121)		0.015
Multiple sclerosis	68.9 (188)	77.6 (156)	84.4 (103)		0.002
Nausea	40.3 (102)	52.1 (98)	59.0 (69)		0.002
Parkinson’s disease	72.1 (191)	81.0 (166)	84.7 (105)		0.008
Persistent muscle spasm	78.3 (231)	86.5 (179)	93.8 (120)		<0.001
Epilepsy	69.2 (182)	77.7 (160)	79.3 (92)		0.043
Terminal illness	77.8 (217)	87.9 (175)	93.0 (119)		<0.001
	**Graduate status**				***p* value**
**Participants’ agreement on the effectiveness of MC on medical conditions**	**Undergraduate**		**Other**		
	**% (*n*)**		**% (*n*)**		
Fibromyalgia	73.0 (356)		87.2 (34)		0.033
Glaucoma	55.5 (241)		33.3 (11)		0.111
Insomnia/Sleeping disorders	79.2 (500)		95.3 (41)		0.004
Persistent muscle spasm	83.3 (490)		95.2 (40)		0.024
Terminal Illness	83.4 (467)		95.7 (44)		0.015
	**Religion**				
**Participants’ agreement on the effectiveness of MC on medical conditions**	**Christian orthodox**		**Non-christian orthodox**		***p* value**
	***n* (%)**		***n* (%)**		
Arthritis	67.6 (361)		89.6 (43)		0.001
Cachexia	68.3 (315)		83.3 (30)		0.040
Nausea and/or vomiting due to cancer treatment	69.1 (394)		81.8 (45)		0.031
Chronic pain	87.4 (563)		96.9 (62)		0.012
Eating disorders	50.5 (268)		78.6 (33)		<0.001
Fibromyalgia	72.2 (350)		95.2 (40)		<0.001
Glaucoma	52.8 (229)		69.7 (33)		0.042
Inflammatory bowel disease	63.1 (305)		88.6 (31)		0.001
	**Field of study**				***p* value**
**Participants’ agreement on the effectiveness of MC on medical conditions**	**Nursing**	**Physiotherapist**	**Speech therapy**	**Other**	
	***n* (%)**	***n* (%)**	***n* (%)**	***n* (%)**	
Cachexia	77.1 (128)	69.4 (125)	65.5 (38)	58.1 (54)	0.014
Eating disorders	51.9 (97)	60.2 (121)	47.1 (33)	43.5 (50)	0.025
Fibromyalgia	81.4 (136)	73.8 (144)	63.6 (35)	68.2 (75)	0.020
Insomnia/Sleeping disorders	78.1 (164)	87.1 (203)	76.3 (61)	74.8 (113)	0.011
Parkinson’s disease	76.9 (143)	86.0 (190)	67.6 (48)	69.8 (81)	0.001
Persistent muscle spasm	82.4 (164)	92.1 (209)	74.3 (52)	78.4 (105)	<0.001
Epilepsy	76.8 (139)	81.1 (163)	64.8 (46)	65.2 (86)	0.002

### 3.4. Participants’ attitudes and beliefs regarding formal MC education

The majority of the participants (*n* = 694, 84.7%) reported that they had never received any formal education on MC during their studies and clinical practice. Additionally, 414 (50.5%) believed that healthcare students should receive formal education on MC laws and regulations during their studies.

### 3.5. Participants’ sources of information on MC

Concerning participants’ sources of information on MC, the most frequently reported sources of information were medical literature (*n* = 446, 54.5%), classroom lectures (*n* = 299, 36.5%), and clinical experience related to care (*n* = 289, 35.3%); fewer participants stated that the main source of information about MC was their personal use (*n* = 58, 7.1%), and cannabis dispensaries (*n* = 53, 6.5%; owners of cannabis shops or employees working in these shops).

### 3.6. Attitudes, beliefs, and knowledge regarding MC in terms of participants’ sociodemographic characteristics

#### 3.6.1. Gender

Female compared to male participants reported more frequency classroom lectures as the main source for information on MC (76.5 vs. 23.5%, *p* < 0.001; Table 3). In contrast, male compared to female participants reported more frequently that they had a family member (13.1 vs. 8.8%, *p* = 0.039) or a friend(s) (28.2 vs. 14.3%, *p* < 0.001) who used MC. Moreover, male compared to female participants expressed more strongly: (a) the intention to recommend MC to their patients (88.0 vs. 83.2%, *p* = 0.046), and (b) that physicians should recommend cannabis for medical therapy (87.6 vs. 80.2%, *p* = 0.005), and (c) that cannabis should be legalized for recreational use (56.8 vs. 38.4%, *p* < 0.001). In contrast, female compared to male participants believed more frequently that using cannabis can be addictive (91.5 vs. 85.9%, *p* = 0.02; Table 4).

**Table 3:** Association between the source of information about medicinal cannabis and participants’ characteristics.

	Classroom lectures		Personal use of recreational cannabis		Personal use of medical cannabis		Cannabis dispensary owners/workers		Medical literature		Friends/Family use of recreational cannabis	
Which sources of information do you use?	% (*n*)	*p*	% (*n*)	*p*	% (*n*)	*p*	% (*n*)	*p*	% (*n*)	*p*	% (*n*)	*p*
Gender		<0.001		<0.01	NSS			0.01	NSS		NSS	
Female	76.5		10.9 (61)				5.0 (28)					
Male	23.5		24.7 (64)				9.7 (25)					
Age		0.019	NSS		NSS			0.014	NSS		NSS	
18–20	37.1 (144)						5.7 (22)					
21–22	41.3 (104)						4.4 (11)					
>23	28.2 (51)						11.0 (20)					
Religion		0.041		<0.05	NSS		NSS		NSS			<0.001
Christian orthodox	37.4 (279)		13.0 (97)								15.8 (118)	
Other	26.7 (20)		37.7 (125)								29.3 (22)	
Graduate status		<0.05	NSS		NSS		<0.05		NSS			0.01
Undergraduate	37.4 (285)						5.2 (41)				18 (137)	
Other	26.9 (14)						23.1 (12)				5.2 (3)	
Field of study	NSS			<0.01		0.022	NSS		NSS		NSS	
Nursing			13 (33)		8.3 (21)							
Physiotherapist			22 (61)		9.7 (27)							
Speech therapy			3.6 (4)		2.7 (3)							
Other			15.1 (27)		3.9 (7)							
Year of study	NSS			0.015	NSS		NSS			0.017	NSS	
First			11.6 (20)						48.3 (83)			
Second			13.8 (22)						13.8 (73)			
Third			17 (39)						17 (39)			
Fourth			19.5 (41)						59.5 (123)			
Fifth or more			0						43.2 (16)			

NNS, non-statistically significant.

**Table 4:** Association between socio-demographic characteristics and participants’ attitudes, beliefs and knowledge on MC.

	Gender		Total	*p* value
	Female	Male		
	% (*n*)	% (*n*)		
++Do you have a family member who uses/had used MC?	8.8 (49)	13.1 (34)	10.1 (83)	0.039
++Do you have friend(s) who uses/had used MC?	14.3 (80)	28.2 (73)	18.3 (150)	<0.001
++Do you have friend(s) who uses/had used recreational cannabis daily or weekly?	41.2 (230)	65.6 (170)	48.8 (400)	<0.001
+Would you recommend MC for your patients?	83.2 (466)	88.0 (228)	84.5 (692)	0.046
+Do you believe that MC physicians should recommend MC as medical therapy?	80.2 (449)	87.6 (226)	82.4 (675)	0.005
+Do you believe that MC there are significant mental health benefits using MC?	76.3 (427)	84.9 (220)	79 (647)	0.003
+Do you believe that MC should be legalized for recreational use?	38.4 (213)	56.8 (147)	44 (360)	<0.001
+Do you believe that MC can be addictive?	91.1 (510)	85.9 (220)	89.1 (730)	0.020
	**Age**			
	**18–20 years old**	**21–22 years old**	**>23 years old**	***p* value**
	**% (*n*)**	**% (*n*)**	**% (*n*)**	
++Do you have a family member who uses/had used recreational cannabis daily or weekly?	11.3 (44)	10.7 (27)	23.0 (41)	<0.001
++Do you have a friend(s) who uses/had used MC?	12.6 (49)	19.4 (49)	31.5 (56)	<0.001
++Do you have a friend(s) who uses/had used recreational cannabis daily or weekly?	45.1 (175)	46.0 (116)	61.8 (110)	0.001
+Do you believe that MC can poses physical health risks?	77.6 (301)	65.1 (164)	67.6 (121)	0.001
+Do you believe that MC can poses mental health risks?	79.9 (310)	67.9 (171)	69.3 (124)	0.001
*Would you recommend MC for your patients?	37.6 (41)	42.2 (46)	20.2 (22)	0.002
*I am prepared to answer patient/client questions about MC	46 (120)	29.5 (77)	24.5 (64)	0.025

	Religion		*p* value
	Christian orthodox	Other	
+Do you believe that marijuana should be legalized for recreational use?	42.9 (319)	91.1 (41)	0.036
+Do you believe that MC can be addictive?	90.3 (672)	77.3 (58)	0.016
+Do you believe that using MC can poses serious physical health risks?	73.3 (546)	54.1 (40)	0.001
+Do you believe that using MC can poses serious mental health risks?	74.8 (557)	64 (48)	0.046
++Do you have a family member who uses/has used recreational cannabis daily or weekly?	12.8 (95)	23.0 (17)	0.016
++Do you have a friend(s) who uses/has used recreational cannabis daily or weekly?	46.2 (344)	77.0 (57)	<0.001
++Would you recommend MC for your patients?	83.9 (626)	93.3 (70)	0.017

+The table presents the *n* and % of participants who answered that they “agree.”

++The table presents the *n* and % of participants who answered “yes.”

^*^Τhe participants who had received formal education about Medical Cannabis.

MC, medical cannabis.

Regarding gender differences on MC benefits, both males and females believed that MC provide significant benefits in physical and mental health. Concerning mental health benefits, more male participants compared to female ones (84.9 vs. 76.3%), agreed that there are significant benefits to using MC (*p* < 0.05). At the same time, both genders believed that the use of MC could pose serious risks to physical and mental health, with female participants agreeing more frequently than males in both cases (physical health: 76.8 vs. 60.6%, *p* < 0.001; mental health: 77.9 vs. 66%, *p* < 0.001; Table 5).

**Table 5:** Association between participants’ gender and beliefs regarding the benefits/risk related to the use of MC.

			Physical health					Mental health		
There are significant benefits using MC	Total		*X* ^2^	OR (95%CI)	*p*	Total		*X* ^2^	OR (95%CI)	*p*
	*n*	%				*n*	%			
Male			1.121	0.9 (0.60–1.41)	0.728			8.06	0.6 (0.49–0.89)	0.005
Disagree	36	13.9				39	15.1			
Agree	223	86.1				220	84.9			
Female										
Disagree	83	14.8				133	13.8			
Agree	477	85.2				427	76.2			
Using marijuana poses serious risks	Total		*X* ^2^	OR (95%CI)	*p*	Total		*X* ^2^	OR (95%CI)	*p*
	*n*	%				*n*	%			
Male			23.00	1.6 (1.35–2.0)	<0.001			13.06	1.4 (1.20–1.81)	<0.001
Disagree	102	39.4				88	34			
Agree	157	60.6				171	66			
Female										
Disagree	130	23.3				124	22.1			
Agree	430	76.8				436	77.9			

The aforementioned differences remained statistically significant even when further statistical analysis was performed in terms of school of study, and gender as independent variables.

#### 3.6.2. Age

In terms of age, the participants were divided into three groups. The first group was between 18 and 20 years old, the second was between 21 and 22 years old, and the third group was over 23 years old. Almost half of the participants in the second group reported that their main source of information about MC was classroom lectures (41.3%) compared to the other two groups (vs. 37.1 and 28.5%, *p* = 0.019 first and third, respectively). In contrast, the third group declared that their main source of information was cannabis dispensary owners and workers compared to the first and the second group (11.0 vs. 5.7% and 4.4%, *p* = 0.014, respectively; Table 3).

Moreover, statistically significant differences were observed in whether they believed that the use of MC could pose health risks. Specifically, the age group between 18 and 20 years reported the highest scores compared with the other age groups in the belief that MC poses physical (77.6 vs. 65.1%, 21–22 years old, and 76.6%, ≥ 23 years old) or mental health risks (79.9 vs. 67.9%, 21–22 years old and 69.3%, ≥ 23 years old, *p* = 0.001; Table 4).

#### 3.6.3. Religious status

In terms of religion, the Orthodox Christian participants (vs. non-Orthodox Christian participants) used classroom lectures as their main source of information (37.4 vs. 26.7%, *p* = 0.041), while the non-Orthodox Christian participants (non-denominational/atheist and Muslim; vs. Orthodox Christian) reported relying on personal experience in using MC/recreational cannabis as their main source of information (37.7 vs. 13%, *p* < 0.05; Table 3). Of the Orthodox Christian participants, 73.3% reported that cannabis can pose serious physical health risks and 74.8% reported that it could pose serious mental health risks, while the non-Orthodox Christian participants exhibited lower percentages (54.1% and 64% respectively; *p* < 0.005). Finally, the non-Orthodox Christian participants agreed that they would recommend MC to their patients (93.3%; *p* = 0.017; Table 4).

Furthermore, over 80% of the non-denominational and atheist participants considered the use of MC as acceptable for specific medical conditions, such as arthritis, cachexia, nausea and/or vomiting due to cancer treatment, chronic pain, fibromyalgia, glaucoma, inflammatory bowel disease, insomnia, sleep disorders, multiple sclerosis, Parkinson’s disease, persistent muscle spasm, epilepsy, and terminal illness (Table 2).

#### 3.6.4. Educational characteristics

##### 3.6.4.1. Level of study

Regarding educational characteristics, statistically significant differences were observed between undergraduate compared to postgraduate participants, who reported classroom lectures as their main information source on MC (37.4 vs. 26.9%, *p < 0.05*), and postgraduate participants, for whom the main source of information on MC were the cannabis dispensary owners/workers (23.1 vs. 5.2%, *p < 0.05*; Table 3).

Furthermore, postgraduate compared to undergraduate participants believed that there were significant mental health benefits associated with the use of MC (94.8 vs. 84.8%, *p* < 0.05), and stated that they would recommend MC to their patients (93.1 vs. 84.1%, *p* < 0.05). Finally, undergraduate compared to postgraduate participants believed that using cannabis can poses serious physical health risks (72.4 vs. 60.3%, *p* = 0.038; Table 6, Part A).

**Table 6:** Association between participants’ academic characteristics and their attitudes, beliefs and knowledge on MC.

Part (a)	Graduate status				
	Undergraduate		Other		*p* value
	% (*n*)		% (*n*)		
+Do you believe that there are significant mental health benefits using MC?	84.8 (647)		94.8 (55)		0.033
+Do you believe that using MC can poses serious physical health risks?	72.4 (551)		60.3 (35)		0.038
++Would you recommend MC for your patients?	84.1 (642)		93.1 (54)		0.042

**Part (b)**	**Field of study**				
	**Nursing**	**Physiotherapist**	**Speech therapy**	**Other**	***p* value**
	**% (*n*)**	**% (*n*)**	**% (*n*)**	**% (*n*)**	
+Do you believe that there are significant mental health benefits using MC?	76.7 (194)	85.6 (237)	76.8 (86)	73.2 (131)	0.008
+Do you believe that marijuana should be legalized for recreational use?	43.7 (110)	55.6 (153)	32.7 (36)	34.1 (61)	<0.001
+Do you believe that marijuana can be addictive?	92.4 (232)	82.6 (228)	97.3 (109)	91.1 (163)	<0.001
+Do you believe that using MC can poses serious physical health risks?	80.6 (204)	60.0 (165)	83.0 (93)	69.3 (124)	<0.001
+Do you believe that using MC can poses serious mental health risks?	81.0 (205)	64.4 (177)	86.6 (97)	70.4 (126)	<0.001
++Do you have friend(s) who uses/had used MC?	13.8 (35)	25.3 (70)	11.6 (13)	20.5 (36)	0.001
++Do you have friend(s) who uses/had used recreational cannabis daily or weekly?	41.9 (106)	58.8 (163)	34.8 (39)	52.8 (93)	<0.001

Part (c)	Year of study					
	First	Second	Third	Fourth	Fifth or more	*p* value
	% (*n*)	% (*n*)	% (*n*)	% (*n*)	% (*n*)	
+Do you believe that there are significant mental health benefits using MC?	66.9 (115)	81.3 (130)	83.8 (192)	81.9 (172)	73.0 (27)	<0.001
++Do you have friends who uses/ has used MC?	11.0 (19)	17.5 (28)	10.5 (47)	21.5 (45)	29.7 (11)	0.023
+Would you recommend MC for your patients?	76.7 (132)	86.9 (139)	88.2 (202)	86.7 (182)	78.4 (29)	0.012
+Do you believe that physicians should recommend MC as medical therapy?	70.3 (121)	88.8 (142)	85.5 (195)	84.8 (178)	78.4 (29)	<0.001
+Do you believe that educational training for MC must be integrated into the academic programs of the health and welfare professionals?	88.4 (152)	95.6 (153)	87.3 (200)	93.8 (197)	81.1 (30)	0.005
+Do you believe that MC should be legalized for recreational use?	30.6 (52)	44.3 (70)	55.0 (126)	42.6 (89)	45.9 (17)	<0.001
+Do you believe that using MC can poses serious mental health risks?	80.8 (139)	76.7 (122)	76.3 (174)	65.2 (132)	70.3 (26)	0.007

+The table presents the *n* and % of participants who answered that they “agree.”

++The table presents the *n* and % of participants who answered “yes.”

MC, medical cannabis.

##### 3.6.4.2. Study field

Participants from nursing studies reported that the main information sources on MC were clinical practice (32%) and experiences with patients/clients (46.2%). Participants from other healthcare study fields reported different sources of information. Meanwhile, participants from physiotherapy studies reported their personal experience with MC (9.7%) or with recreational cannabis (22%) as their main source of information. Furthermore, compared with participants from other study fields (nursing students 76.7%, speech therapist students 76.8%, and others 73.2%), physiotherapy students (85.6%), believed more strongly that there were significant mental health benefits associated with MC use (*p* = 0.008). On the other hand, almost all participants from speech therapy field (97.3%) compared to the other study group participants (nursing students 92.4%, physiotherapist students 82.6%, and others 91.1%), reported that cannabis can be addictive (*p* < 0.001) and that its use can pose serious physical or mental health risks (*p* < 0.001; Table 6, Part B).

### 3.7. Differences between formal education about MC and participants’ personal and academic characteristics, and attitudes, beliefs, and knowledge

Only a small number of participants (15.4%, *n* = 126) received the education in their curricula/clinical setting.

Regarding age variations, those aged over 23 years (13.9%) were less likely to receive formal education on MC through their curricula or clinical setting compared to participants aged 18–20 years (11.8%) and those who were aged 21–22 years old (21.8%; *p* = 0.002).

Moreover, the participants who had friend(s) (24.7%) or family member**s** (22.9%) who used MC had received more frequently formal education than those who had no friends (13.2%; *p < 0*.001) or had no family members (14.5%; *p < 0*.005) who used MC.

With regard to educational characteristics, fourth-year participants (19.3%) reported more frequently that they had received formal education than the participants who were students in a different year of study (first year: 5.8%, second year: 16.1%, third year: 17.8%, and fifth year: 16.2%; *p* = 0.001). Additionally, in terms of field of study, participants from physiotherapy had the highest proportion on having received formal education (15.9%; 44/277), followed by the participants from nursing (15.5%; 39/252), and speech therapy (2.7%, 3/112) studies (*p* < 0.001).

Furthermore, participants with 1–5 years of work experience had received more frequently formal education on MC (23.9%) than participants with more than 5 years of experience (19.3%) or those without any experience (12.2%; *p* < 0.001).

Moreover, 259 participants considered him/her self academically prepared to answer healthcare service users’ questions about MC. Furthermore, of those who had received formal education on MC approximately 23.3% reported that they were prepared to answer patients’ questions about MC, surprisingly less than those who had not received formal education related to MC (76.7%; *p* < 0.001). However, more than one out of 10 (13.4%) participants who had received formal education were neutral about whether they were prepared to answer patient/client questions about MC (Table 7).

**Table 7:** Association between participants’ formal education on MC, their attitudes, beliefs and knowledge on MC and socio-demographic characteristics.

Received any formal education about MC	Yes		No		*Χ* ^2^	DF	*p*
	Total		Total				
	*N*	%	*N*	%			
Age					12.43	2	0.002
18–20	46	11.8	341	88.2			
21–22	55	21.8	197	78.2			
<23	25	13.9	155	86.1			
Religious					0.03	1	0.860
*Other*	12	16	63	84			
*Christian orthodox*	114	15.4	630	84.6			
Which year are you studying?					19.32	4	0.001
First year	10	5.8	163	94.2			
Second year	25	16.1	135	83.9			
Third year	41	17.8	195	82.2			
Fourth year	44	19.3	168	78.7			
Fifth year and more	6	16.2	31	83.8			
What is your field of study					22.94	3	<0.001
Nursing	39	15.5	213	84.5			
Physiotherapists	44	15.9	233	84.1			
Speech therapy	3	2.7	109	97.3			
Other	40	22.5	138	77.5			
Past years of work experience					15.98	2	<0.001
No previous experience	71	12.2	512	87.8			
1–5 years	49	23.9	156	76.1			
>5 years	6	19.3	25	80.7			
I am prepared to answer patient/client questions about MC					17.27	1	<0.001
Agree	60	23.3	199	76.7			
Disagree	20	9	202	91			
Neutral	46	13.4	292	86.6			
Ι would recommend MC for my patients					0.68	1	0.421
Agree	109	15.7	586	84.3			
Disagree	16	12.9	108	87.1			
Physicians should recommend cannabis as a medical therapy					0.44	1	0.507
Agree	19	13.4	569	84.4			
Disagree	105	15.6	123	86.6			
							
Total	126	15.4	693	84.6			

Received any formal education about MC	Yes		No		*Χ* ^2^	DF	*p*
	Total		Total				
	*N*	%	*N*	%			
There are significant physical health benefits using medical cannabis					1.25	1	0.262
*No*	14	11.6	104	88.1			
*Yes*	111	15.9	588	84.1			
There are significant mental health benefits using medical cannabis					0.02	1	0.926
*No*	26	15.2	145	84.8			
*Yes*	99	15.3	547	84.7			
Educational training for medical cannabis must be integrated into the academic programs of the health and welfare professions					1.40	1	0.235
*No*	15	20	60	80			
*Yes*	110	18.8	632	85.2			
Educational training in the use of medical cannabis should be integrated into the practice / clinical practice requirements of students in health and social care professions					0.16	1	0.745
*No*	23	16.1	120	83.9			
*Yes*	101	15.0	572	85.0			
Health and welfare professionals should have formal training on the medical cannabis before recommending it to someone who is being treated					0.95	1	0.329
*No*	10	20	40	80			
*Yes*	114	14.9	652	85.1			
Marijuana should be legalized					0.10	1	0.745
*No*	62	13.7	391	86.3			
*Yes*	62	17.3	297	82.7			
Do you have a family member who uses/has used recreational cannabis daily or weekly?					1.25	1	0.263
*No*	104	14.8	599	85.2			
*Yes*	21	18.9	90	81.1			
Do you have a family member who uses/has used MC?					4.03	1	0.045
*No*	106	14.5	625	85.5			
*Yes*	19	22.9	64	77.1			
Do you have a friend who uses/has used MC					12.69	1	<0.001
*No*	87	13.2	573	86.8			
*Yes*	38	24.7	116	75.3			
Do you have a friend who uses/has used medical cannabis for recreational purposes daily or weekly?					0.79	1	0.374
*No*	59	14.3	355	85.7			
*Yes*	66	16.5	334	83.5			
Marijuana can be addictive					0.37	1	0.543
*No*	15	82.6	71	82.6			
*Yes*	109	15.1	620	85			
							
Total	126	15.4	693	84.6			

## 4. Discussion

To the best to our knowledge, this is the first study to describe university healthcare students’ attitudes, beliefs, and knowledge regarding MC in the Republic of Cyprus, and among the few on the subject internationally. Specifically, we examined the association of sociodemographic (gender, age, religious status) and educational characteristics (level of study and study field) with healthcare students’ attitudes, beliefs, and knowledge on MC. Previous studies on the use of MC mainly focused on prescribers or on a single disease category (27, 28). Τhus, the present study adds to existing national and international literature by providing new data on the attitudes, beliefs, and knowledge of healthcare students in the Republic of Cyprus, while one of the strengths of the study is its methodological rigor, as well as the relatively high response rate in terms of the number of students studying in the given time period.

Our results showed that *gender* was significantly associated with participants’ attitudes and beliefs regarding the use of MC. Male participants, compared with female participants, expressed stronger willingness to recommend MC to their patients and they more strongly believed that physicians should recommend cannabis for medical therapy. Although both males and females believed that MC provides significant benefits in physical and mental health, they also believed that MC use could pose serious risks to physical and mental health; yet, female participants agreeing more frequently than male participants in the above. Additionally, our results revealed that male participants used cannabis for recreational purposes more frequently, and were more concerned about the mental health benefits of using MC than female participants. This finding may highlight the link between the personal experience of cannabis use and its perceived benefits to mental health during students’ life (25). Previous studies have shown contradictory results regarding the link between gender and MC related attitudes and knowledge (5, 29). Evidence from international research supports a higher frequency of cannabis use among male healthcare students than among female students (2, 11, 21). These results have been associated with sociocultural explanations, including factors related to gender roles and biological and psychological patterns (21). Sokratous et al. (21) found that female nursing students had more knowledge of the benefits of MC and more positive attitudes toward the need for formal MC related education than male nursing students. According to previous studies, male students use cannabis more frequently than female students do, which may be related to the fact that male participants’ knowledge and beliefs are based on their personal experiences (21, 22, 30). Our results showed that both genders believed that marijuana use can pose serious risks to mental and physical health.

Moreover, the present results supported the association between the participants’ attitudes, beliefs, and knowledge regarding MC and their *age*. Almost half of the participants of the group aged 21–22 years reported that their main information source about MC was classroom lectures or clinical practice, compared to the other age groups. On the contrary, those aged over 23 years, reported that their main source of information was cannabis dispensary owners and workers compared to the participants who were aged between 18 and 22 years. These results may be related to new curriculum development, educational changes, and policy decisions related to cannabis use for medical purposes in the Republic of Cyprus in the last 2 years.

Our results showed that *religion* marked a statistically significant difference, with non-Orthodox Christian participants being likelier than participants of other religions to recommend MC to their patients. On the other hand, Orthodox Christian participants reported that cannabis can pose serious physical and mental health risks in a higher percentage than non-Orthodox Christian participants. In the literature, religiosity refers to participation in an organized religion and has been identified as a factor linked to prevention and treatment of substance use (18). However, little attention has been paid to the association between religiosity and university healthcare students’ knowledge, attitudes, and beliefs about MC (29).

Furthermore, concerning the *level of study*, postgraduate participants believed that there are significant mental health benefits associated with the use of MC, compared with undergraduate participants, and reported that they would recommend MC to their patients in a higher percentage. On the other hand, undergraduate participants believed that using MC can pose serious physical health risks, which probably reflects a lack of experience in the clinical setting, a greater lack of friction with patients, and the absence of education in university curricula regarding the use of MC.

In terms of the *field of study*, the participants from the physiotherapy departments agreed the most on the MC clinical benefits compared to other participants (e.g., nursing students). These results may be explained by the fact that in physiotherapy departments, MC-related courses are offered more often than in other study fields (e.g., nursing) during healthcare studies (5, 21). It could be hypothesized that students are more willing to express their attitudes toward cannabis when relevant topics are openly discussed during classes. These findings highlight the need for curricula designed to inform students about the use of cannabis for students to be adequately prepared to work with patients who may use MC.

Yet, we need to underline that since medical cannabis use was legalized in 2019 in the Republic of Cyprus, the number of physiotherapy student participants having personal experience with MC is quite high considering the short time after the legalization. One explanation for this may arise from the participants’ experiences and knowledge about MC they had before the legalization of MC for therapeutic use. Another explanation may be related to the experiences of others (family/relatives, patients) with MC before legalization. It is worth noting that it was possible to find and use MC event before legislation in the Republic of Cyprus, coming from the free market, or event from the internet market.

### 4.1. Participants’ attitudes, beliefs, and knowledge about MC and the treatment of specific disorders

Our results showed that a vast majority of participants support the use of MC. Additionally, it was found that the participants hold moderate knowledge of the risks and benefits of patients’ use of MC. Our results also showed that several participants considered themselves academically prepared to answer patient/client questions on MC and reported satisfactory confidence when discussing MC benefits for specific disorders with their patients. Participants who had friend(s) or family member**s** who use/had used MC considered themselves academically prepared to answer patient/client questions on MC and reported that they had received more formal education compared with those who had no friend (s) or family members who use or had used MC. In particular, a high percentage of participants believed that the use of MC was acceptable for patients with Alzheimer’s disease, arthritis, cachexia, nausea and/or vomiting due to cancer treatment, chronic pain, eating disorders, fibromyalgia, glaucoma, insomnia or other sleep disorders, mental health disorders, multiple sclerosis, nausea, Parkinson’s disease, persistent muscle spasm, epilepsy, and terminal illnesses. At the same time, they did not believe that MC could be useful for patients with AIDS or HIV. Previous studies on a similar population in Cyprus are in agreement with these results (21, 22). More specifically, previous studies on nursing students (21) and nurses and midwives (22) have shown a lack of knowledge of the benefits of MC and less confidence in discussions about MC. Furthermore, in this study, we observed a strong association between participants who would recommend MC to their patients, assuming that physicians would recommend MC as medical therapy.

Previous research evidence from the literature have shown that health practitioners have insufficient theoretical and clinical knowledge of the use of MC and its benefits (5, 31). At the same time, although scientific evidence remains scarce, participants acknowledged that the therapeutic potential of cannabis may be explained by the fact that they personally know people who use cannabis and, thus, may be aware of such positive effects (22). These findings highlight the significant need for curricula designed to inform students about the use of MC for students to be adequately prepared to work with patients who may use this substance (32). The participants who believed that there are significant physical health benefits to using MC and that educational training in its use should be integrated into clinical practice requirements of students in health and social care were more likely to recommend MC to their patients.

In our study, the participants who reported that health and welfare professionals should have formal training in MC before recommending it to someone being treated were likelier to recommend MC to their patients. These results are supported by other research evidence from national and international literature, which highlights the necessity of providing formal education on MC among healthcare professionals (10, 33).

### 4.2. Participants’ attitudes and beliefs regarding formal education on MC

Regarding formal education in our study, a vast majority of the participants strongly believed that educational training in the use of MC should be integrated into clinical practice, and eight out of 10 participants supported the idea that physicians should recommend cannabis for medical therapy. The students who participated in this study reported, in high percentages, that they had never received any formal education on MC during their study and clinical practice. Only a small number (15.4%, *n* = 126) of participants received formal education about MC in their curricula and clinical setting. These groups of students supported the idea that they felt academically and clinically prepared and ready to answer patient/client questions regarding the use of MC.

The participants herein believed that they should receive formal education about MC laws and regulations during their studies, and the most frequently reported source of information was medical literature studies. The present results support that formal education and clinical experience are important and catalytic factors in being academically and clinically prepared to answer patient/client questions regarding the use of MC.

In conclusion, this study provides useful information for curriculum development, educational changes, and policy decisions related to cannabis use for medical purposes in Cyprus. The results showed that the majority of healthcare students in Cyprus favored the use of MC. However, the participants reported a lack of knowledge and recommended additional evidence-based research and education to enhance their knowledge of the use of MC. Therefore, we recommend the implementation of formal education on MC among healthcare students in Cyprus during their study and clinical training. Furthermore, it is important to include MC-related theoretical and clinical/laboratory courses during studies and clinical practice. The results of our study are in line with the guidelines of the National Council of State Boards of Nursing (NCSBN), United States, which supported that all healthcare professionals and students (e.g., nursing students) should be trained at least in the basic knowledge regarding the use of cannabis for patient safety, and specific techniques in approaching their patients without judging their choice of treatment (34).

However, this finding requires further investigation. One of the weaknesses of the present study regards the absence of medical and psychology students, which influences the generalizability of the present findings to the entire population of health professional students. Including students from all health allied sciences would increase the range and depth of the present findings. However, the present findings still provide a rigor trend concerning the attitudes, beliefs, and knowledge regarding MC of a great proportion of healthcare students in the Republic of Cyprus, also supported by the high response rate and the methodological integrity of the present sample.

An additional limitation of the study was that the questionnaire used herein did not allow participants to report if they had any formal education on pharmacology. Instead, the present questionnaire included only one item asking the participants if they had received or not any formal education on MC. As a result, it was not possible to collect any data on the training regarding pharmacology, which would provide data on the clinical background and experience of the responders on pharmacological interventions. Future studies need to address this limitation and include an item on the kind of education the participants have received regarding both pharmacology in general and MC interventions, as well.

Moreover, additional limitations of our study include a lack of triangulation with qualitative data and a possible underestimation of the actual frequency of positive attitudes toward MC. More importantly, the cross-sectional nature of the study does not permit any inference regarding the direction of the observed association between the use of MC and healthcare students’ attitudes, beliefs, and knowledge. Nevertheless, in our study, the large sample size and use of an appropriate and robust instrument allowed for a more accurate estimation of healthcare students’ attitudes, beliefs, and knowledge regarding the use of MC.

## Data availability statement

The original contributions presented in the study are included in the article/Supplementary material; further inquiries can be directed to the corresponding author.

## Ethics statement

The study was reviewed and approved by Cyprus National Bioethics Committee (Ref. No 2019.01.155). The role of the review Bioethics Committees was: (1) Contribute to safeguarding the dignity, rights, safety, and well-being of all research participants. (2) Provide independent, competent, and timely review of the ethical aspects of proposed studies. (3) Be responsible for carrying out the review of research proposal before the commencement of the research. Additionally, the study was approved by university research committees. All methods were carried out in accordance with the relevant guidelines and regulations of the aforementioned committees. The participants were informed about the purpose of the study and the data collection procedures prior to providing their consent. All participants agreed to participate and informed consent was obtained from all subjects and/or their legal guardians. Participation in the study was voluntary and anonymous in order to guarantee confidentiality. The participants provided their written informed consent to participate in this study.

## Author contributions

The present study was jointly designed by SS, MM, and MK. Each author made substantial contributions to the conception, design, analysis, and interpretation of the data and was involved in drafting and/or critically revising the manuscript for important intellectual content. All authors contributed to the article and approved the submitted version.

## Funding

The present study was partially funded by the Cyprus University of Technology (internal funding code = 319).

## Conflict of interest

The authors declare that the research was conducted in the absence of any commercial or financial relationships that could be construed as a potential conflict of interest.

## Publisher’s note

All claims expressed in this article are solely those of the authors and do not necessarily represent those of their affiliated organizations, or those of the publisher, the editors and the reviewers. Any product that may be evaluated in this article, or claim that may be made by its manufacturer, is not guaranteed or endorsed by the publisher.
